# Land-use stress alters cuticular chemical surface profile and morphology in the bumble bee *Bombus lapidarius*

**DOI:** 10.1371/journal.pone.0268474

**Published:** 2022-05-13

**Authors:** Florian Straub, Jonas Kuppler, Martin Fellendorf, Miriam Teuscher, Juliane Vogt, Manfred Ayasse

**Affiliations:** 1 Institute of Evolutionary Ecology and Conservation Genomics, Ulm University, Ulm, Germany; 2 Chair for Terrestrial Ecology, Technical University of Munich, Freising, Germany; 3 Natura 2000-Station Unstrut-Hainich/Eichsfeld, Hörselberg-Hainich, Germany; USDA-ARS Southeast Area, UNITED STATES

## Abstract

Pollinators and other insects are currently undergoing a massive decline. Several stressors are thought to be of importance in this decline, with those having close relationships to agricultural management and practice seemingly playing key roles. In the present study, we sampled *Bombus lapidarius* L. workers in grasslands differing in their management intensity and management regime across three different regions along a north-south gradient in Germany. We analyzed the bees with regard to (1) their cuticular hydrocarbon profile (because of its important role in communication in social insects) and amount of scent by using gas chromatography and (2) the size of each individual by using wing distances as a proxy for body size. Our analysis revealed changes related to land-use intensity and temperature in the cuticular scent profile of bumble bees. Decreasing body size and increasing total scent amount were explained by an interaction of land-use intensity and study region, but not by land-use intensity alone. Thus, land-use intensity and temperature influence intracolonial communication and size, both of which can have strong effects on foraging. Land management and climate are therefore probably detrimental for colony maintenance and the reproductive success of bumble bees.

## Introduction

During the past few decades, a massive decline in species has occurred world-wide [1]. Insects and other pollinator species are particularly badly affected [2–4]. A decline of one third of insect species in only 10 years has been demonstrated by a long-term study embedded in the Biodiversity Exploratories Project, Germany [4]. The reasons and drivers of this decline include limited food and nesting resources, pathogens and parasites, climate change, and an intensification of land-use [2, 5]. The last-mentioned factor in agricultural areas has been shown to have especially negative effects on various taxa [6–8].

Most of Europe is currently covered with intensively managed and artificial landscapes with only a few small areas remaining natural [9, 10]. The type of land management, e.g., conventional or organic farming practice, as well as the number of non-crop habitats have an effect on the number of bee species and the abundance of bees [11, 12]. However, this long-held assumption of conventional farming being a risk per se has been challenged [13]. The diversification of crop-land and reduced field sizes have been suggested as being a much more powerful tool for promoting biodiversity, even in conventional farming, than organic certification alone [12, 13]. Furthermore, floral resources in a more intense farming setting can be beneficial for pollinators [14], which are affected by farming practice [14, 15]. Such effects affect bees, especially at the regional scale [16]. However, bees respond differently to land-use, and their sensitivity to land-use is influenced by various traits such as the duration of the flight season and flight range [14]. Land-use involves various parameters, e.g. the mowing of fields, the grazing of livestock, and the application of fertilisers and pesticides, all of which pose risks to insects [10, 15, 17–19]. Pesticide treatment with neonicotinoids, for example has been shown to impair communication in insects [20, 21]. Sexual communication and host finding in a parasitoid wasp are disrupted after imidacloprid treatment [20], whereas antennal sensitivity to floral scent compounds is reduced in solitary bees treated with clothianidin [21]. Bees strongly depend on grasslands in their neighbourhood [22]. The homogenization of grasslands leads to species-poor habitats, whereas a high diversity and abundance of flowering plants positively affects pollinator diversity and flower visitation behavior [7, 23, 24]. For bees, a high diversity of plants is crucial, because they visit flowers not only to gain nectar as an important source of carbohydrates, but also to collect pollen as a protein food source for their larvae [25]. Indeed, the effects of a poor diet are multifold. Pollen diversity and quality directly affect the colony development, the reproduction, and the physiology of bees [11, 26–28]. Furthermore, pollen provisions and access to pollen are directly linked, with any decreases in these two factors being shown to result in reduced body size, a key fitness component in pollinators [26, 29–31].

Pollinator size can affect both foraging and colony maintenance. Bumble bees are known to display alloethism, whereby individuals with different sizes perform different tasks in the colony [32]. Large workers usually go out foraging, while smaller ones stay in the nest. Size differences in foragers have effects on: foraging distance, the amount of food carried, flower handling, and thermoregulation [32–35]. Pollinators that forage over large distances invest in flight muscles [34]. The size of pollinators and their muscle volume are therefore directly linked to dispersal and foraging distances: larger foragers can fly greater distances to find suitable food sources than can smaller ones [32–34, 36]. The latter with their smaller foraging range are however more sensitive to local land-use intensity [14]. Larger bees are thought to forage more for nectar and, in total, carry more food than smaller ones [31, 33]. Despite the more limited extent of foraging by small individuals, they have been shown to handle flowers faster and more effectively than big ones [37]. Smaller individuals show poorer thermoregulation [35]. Thus, larger individuals are better adapted to cooler temperatures and are less reliant on warm weather for foraging trips [35, 38]. Size also has an effect on communication or at least on the finding of host plants. For example, bigger bumble bee workers, which have a higher number and density of olfactory sensilla on their antenna, show a higher sensitivity than small bees when exposed to *Jasminum grandiflorum* L. scent [39].

Cuticular lipids fulfill two main functions in insects: waterproofing and communication. The major classes of compounds identified are cuticular hydrocarbons (CHCs), various types of esters, aldehydes and fatty acids. Among the CHCs there are n-alkanes with mainly odd numbers of C-atoms, methyl-branched compounds, and unsaturated hydrocarbons [40–43]. In terms of the anti-desiccation response and thus waterproofing in insects, the CHC profile changes at higher temperatures toward compounds with higher melting points in order to prevent water loss [42, 44]. With regard to communication, the role of cuticle lipids in bumble bees is highly variable. Intracolonial communication is involved in nestmate recognition [40, 45, 46] and alarm behavior [46] and plays an essential role in task allocation and the regulation of worker reproduction [41, 47]. The cuticle lipid profile also reflects physiological changes within an individual bumble bee and thus allows the recognition of task performance, dominance and fertility status [48, 49]. CHC profiles tend to be species-specific, whereas the variation in the nest wax odors at the colony entrance provides the bumble bee *Bombus terrestris* L. with information concerning nest identity and prevents colonies from exploitation by non-nest individuals [45, 46]. Population-specific variation in scent bouquets are also known to lead to dialect-like differences [50, 51]. Cuticular lipids and hydrocarbons are extremely important factors in chemical communication and colony maintenance. Thus, stress-induced changes can cause the disruption of the social structure in a colony or the loss of queen dominance. However, little is known about the links between the various stressors and their effects on the chemical surface profile of bees.

In this study, we have therefore investigated the effects of land-use intensity, of the three Biodiversity Exploratory regions, and of air temperature on the cuticular lipid profile and the size of bumble bees. Because little is known about the relationship between land-use stress and chemical communication [20, 21], we sought new insights into the various effects on pheromones on the cuticle surface, since communication via pheromones is crucial for colony maintenance in social insects [41]. We have used *Bombus lapidarius* as a model species, since it is one of the most commonly occurring wild bee species in Europe [25, 52, 53] and is indispensable for pollination. Our aim has been to answer the following questions:

- Does the scent bouquet of *B*. *lapidarius* change in grasslands with different land-use intensity?
- Does land-use intensity have an effect on the size of workers?
- Does the study region influence the cuticular surface odor and size of *B*. *lapidarius* workers?

## Materials and methods

### Study design

Our project took place within the framework of the Biodiversity Exploratories Project (DFG priority program 1374), which provides a huge research platform for interdisciplinary research in Germany [54]. We examined experimental plots (EPs) with a size of 50×50 m in grasslands within each of the three distinct exploratory regions (henceforth called “region”): the Schorfheide-Chorin in the north-east of Germany, the Hainich-Dün in Central Germany, and the Schwäbische Alb region in the south-west. All EPs are managed by the local owners and farmers. The plots are embedded in real-world management and thus vary in their farming intensity, which leads to a land-use gradient among the EPs of each region. The land-use intensity index (LUI) is calculated as the sum of the three components of grazing (livestock units days of grazing ha^−1^ year^−1^), mowing (frequency of mowing per year), and fertilization (kg nitrogen ha^−1^ year^−1^) [55]. The LUI and thus the degree of anthropogenic influence can range from highly extensive plots such as juniper heaths, grazed by sheep for only a few days per year, to highly intensive meadows and mown pastures that are heavily fertilized and mown up to three times a year, or to a combination of all three components in an intensive manner (S1 Table) [54]. Experimental plots between these extremes are usually only mown once or twice a year and can have additional low grazing pressure or small amounts of fertilization [54]. Furthermore, the EPs can differ in their plant diversity as a result of land-use intensity [6, 8]. Values for the environmental variables, e.g., temperature, included in the analyses were obtained from the Biodiversity Exploratories data platform *BExIS*.

**Fig 1:**
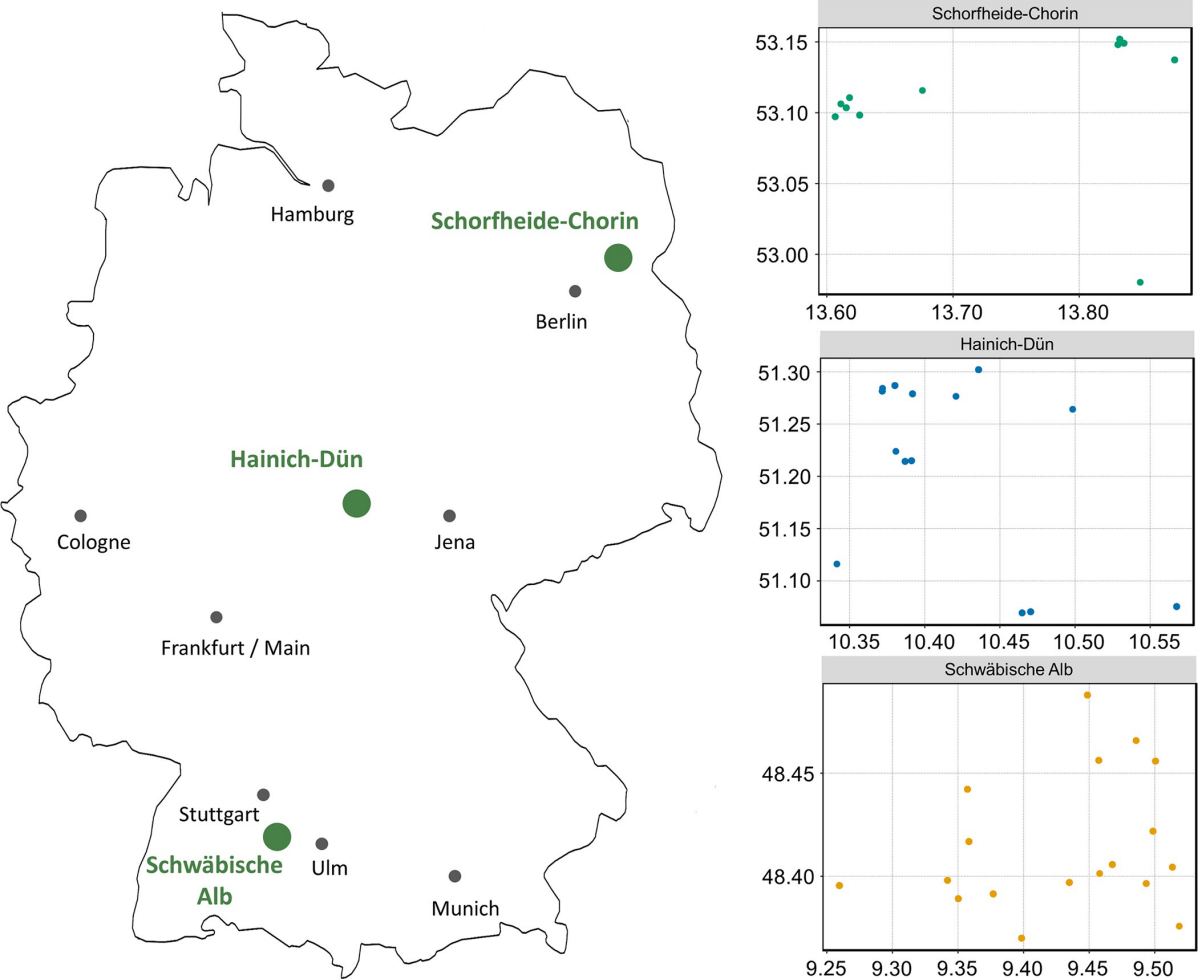
Map of all 42 experimental plots (EPs) in which *B*. *lapidarius* workers were sampled within the Biodiversity Exploratories Project and their location in Germany (Made with Natural Earth. Free vector and raster map data @ naturalearthdata.com). Each dot represents an individual EP.

In total, we sampled 307 individuals from 42 different experimental plots (Schwäbische Alb: 18 EPs, N = 158; Hainich-Dün: 13 EPs, N = 76; Schorfheide-Chorin: 11 EPs, N = 73) in all three regions (Fig 1).

### Study species

For the chemical analysis of surface odors and the measurement of morphological traits, we used *Bombus lapidarius* (LINNAEUS, 1758), which is a common and widespread bumble bee species in Europe and which is classified as being of least concern according to the red list of threatened species [25, 53, 56]. It is a polylectic bee species and forages for nectar and pollen on various plant families, but has a clear preference for several Fabaceae species and *Centaurea* spp. [52]. Using insect nets, we caught *B*. *lapidarius* workers in grasslands under varying management regimes within the Biodiversity Exploratories Project by using requisite-based variable transect walks, which covered the most attractive resource patches [23, 57]. After being caught, individual bees were transferred to 2 mL Eppendorf® tubes and stored in cooling bags filled with cool packs. All individuals were caught between June and July 2018 during a total of 19 sampling days. Bumble bees were sampled on days under suitable weather conditions without rain and heavy winds. We did not observe any variation in bees caught on the different days, the only variation of bees caught was attributable to the number of plots sampled per day. To prevent the unnecessary killing of erroneously caught bees, we identified all bees in the field to species level. After transportation to the laboratory, the bees were freeze-killed and stored at - 40°C until being further processed. All necessary permits for the described study were obtained by the responsible state environmental offices of Baden-Württemberg (55-8/8848.02–07), Thüringen (UH: 10122-17-301; NDH: 364.622/0054-17; EA: 63.2/15.02.11-bio_expl2017.2; EIC: 001-04-18/6-85/uni-München/Biodiversitäts-Exploration; KYF: III.3.3–364.53.1/2018-06-01_BiodivExpl_Ergänz_Arthropoden), and Brandenburg (LFU-N1-4743/128+5#32246/2018), which complied with all relevant regulations.

### Collection of cuticle surface extracts and chemical analysis

Bees were thawed for 4 min prior to scent extraction and individually rinsed for 2 min in 1 mL n-pentane (SupraSolv, 99.9%, Supelco) to extract compounds from their cuticle surface. Before chemical analyses, solvent extracts were concentrated to a final volume of 300 μl by using a gentle stream of nitrogen. As an internal standard, 10 μl dodecane (C12) was added (99%, Sigma, Germany, stock solution: 100 μg/mL in n-hexane) for quantitative analysis.

All chemical analyses were performed on a gas chromatograph (Agilent 7890A, Agilent Technologies, Waldbronn, Germany) with a DB-5 capillary column (30 m × 0.25 mm inner diameter, J&W) and a flame ionization detector (FID). Hydrogen at a constant flow of 2.0 mL/min was used as a carrier gas. One microliter of the respective extract was injected splitless into the gas chromatograph at an injector port temperature of 310°C. After an initial time of 1 min at 50°C, the oven temperature increased continuously by 10°C/min to a final temperature of 310°C and held at that temperature for 35 minutes resulting in a total working time of 62 minutes.

### Wing measurements

After cuticle surface extraction, the forewings of each individual were cut off and mounted on microscopic glass slides (76 × 26 mm, VWR International, Radnor, USA). A second glass slide was placed on top of the wings, which were thus flattened, in order to improve the quality of the subsequently taken photographs. We used an Axiocam 105 color microscope camera (Zeiss, Germany) mounted on a Stemi 508 stereo microscope (Zeiss, Germany) to photograph each individual wing. For photography, we employed the transmitting light source of the stereo microscope in order to avoid any reflections on the wings. As a proxy for size, we measured the distance from the proximal end of the first cubital cell to the distal end of the third cubital cell [53] by means of the analytical software ZEN 3.2 (blue edition, Zeiss). This size was also used to normalize the absolute scent amount on the surface of each individual bumblebee.

## Statistical analysis

All statistical analyses, Linear Mixed-Effects models (LMM), and redundancy analysis (RDA) were performed in R (version 3.5.2) [58]. For the analysis of the chemical surface extracts, we used the peak area of the internal standard dodecane to quantify and calculate the absolute and relative amounts of each single compound. Furthermore, the total absolute scent amount per individual was standardized for size by dividing it with the size of each individual followed by a log-transformation. To obtain the impact of land-use and environmental factors on the chemical surface profile, we ran a distance-based Redundancy Analysis (dbRDA) based on Bray-Curtis dissimilarities by using the function *capscale* from the *vegan* package (version 2.5–6) [59]. The land-use intensity index LUI, ambient air temperature (mean temperature of the past 28 days prior to sample date), and experimental plot EP (“Plot”) were set as explanatory variables. We used the *ordistep* function with backward selection to select the best fitting model. The following model was obtained:

"ScentProfile"∼"LUI"+"Airtemperature"+"Plot"

Before the analysis, our chemical compound matrix was square-root-transformed, followed by a Wisconsin double-standardization in order to emphasise environmental variables. Since the function *capscale* cannot analyze datasets with zeros in rows, we added a very small number (0.000001) to every compound value. To analyze the effects of land-use management and environmental factors on size and on absolute scent amount per unit size of bumble bee, we calculated linear mixed-effect models (LME) by using the *lmer* function from the *lme4* package (version 1.1–23) [60]. We set the land-use intensity index LUI and region as fixed factors and plot as a random factor. Whenever necessary, we ran a post hoc test by using the function *glht* (General Linear Hypotheses) from the *multcomp* package (version 1.4–16) [61]. All model assumptions were validated using the *DHARMa* package (version 0.4.3) [62] and were sufficient.

## Results

### Effects on chemical surface compounds

#### Chemical surface profile

**Fig 2:**
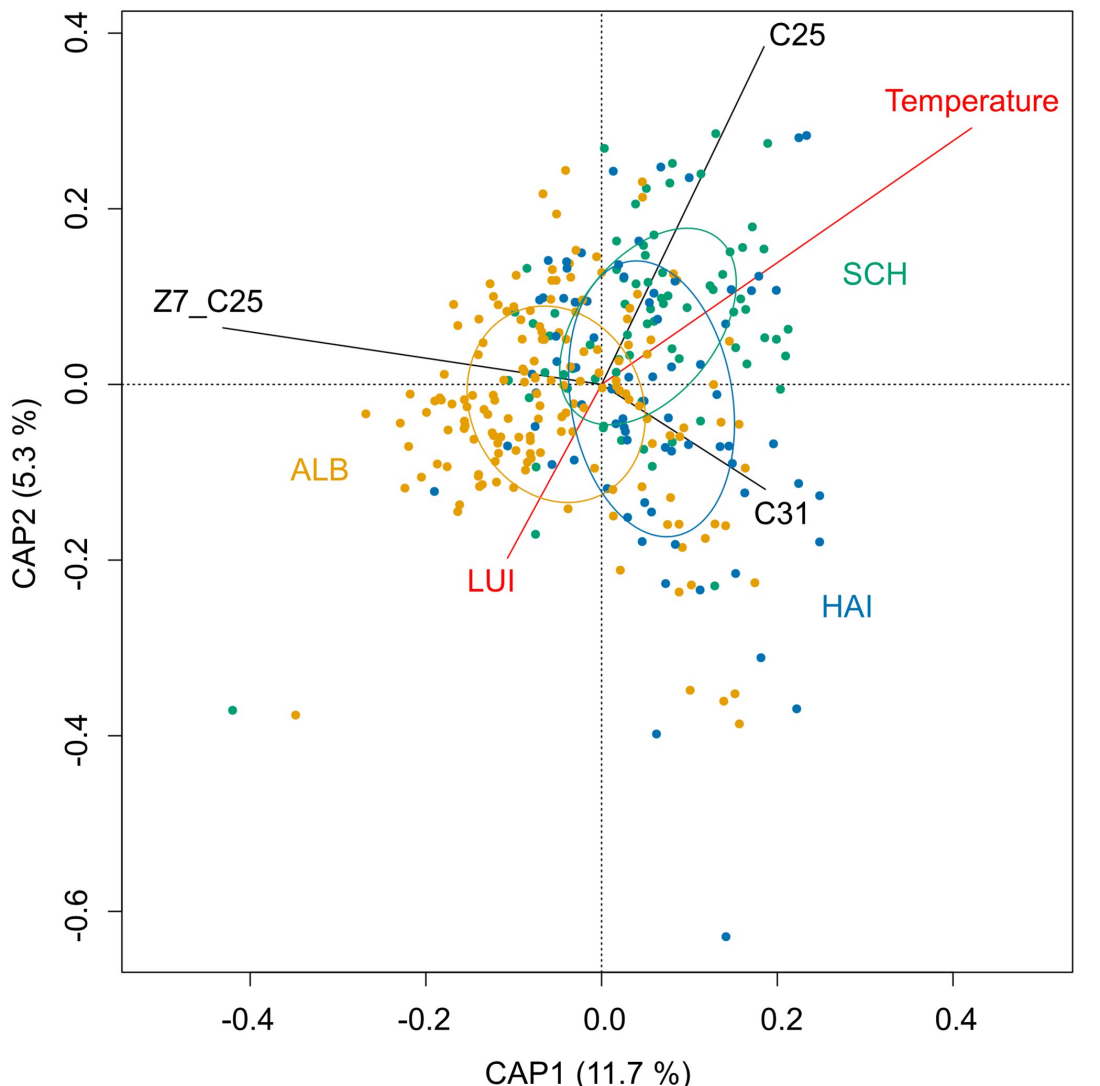
Correlation tri-plot of the first two axes obtained from the distance-based redundancy analysis (dbRDA). The graph shows the relationship between the chemical scent bouquet of lipids on bumblebees and important environmental parameters. Sites are shown as individual plots and ellipses for region groups (ALB: Schwäbische Alb, HAI: Hainich-Dün, SCH: Schorfheide-Chorin). The explanatory variables temperature and land-use intensity (LUI) are shown as red lines, the three scent compounds with the highest loadings in CAP1 and CAP2, namely (Z)-7-Pentacosene (Z7_C25), Pentacosane (C25), and Hentriacontane (C31), are shown as black lines.

In the analysis of the chemical compound data, we identified 49 chemical compounds (S2 Table) known from previous studies to play a role in nest communication [45, 48]. RDA showed that environmental variables explained 31.5% of the variation in the scent profile, with axis 1 explaining 11.7% and axis 2 explaining 5.3% of the variance. The first three canonical axes CAP1 –CAP3 together explained 62.4% of the variation in chemical scent profile. Amongst the three chosen variables, plot was the most important (ANOVA_plot_: = 319.95, N = 307, p < 0.001) followed by LUI (ANOVA_LUI_: = 13.18, N = 307, p < 0.001, Fig 2) and ambient temperature (ANOVA_temperature_: = 5.20, N = 307, p < 0.05, Fig 2). The three scent compounds pentacosane (C25), (Z)-7-pentacosene (Z7_C25), and hentriacontane (C31) showed the highest loadings in CAP1 and CAP2 and contributed most in the variation of scent profile (S3 Table).

#### Absolute scent amount

**Fig 3:**
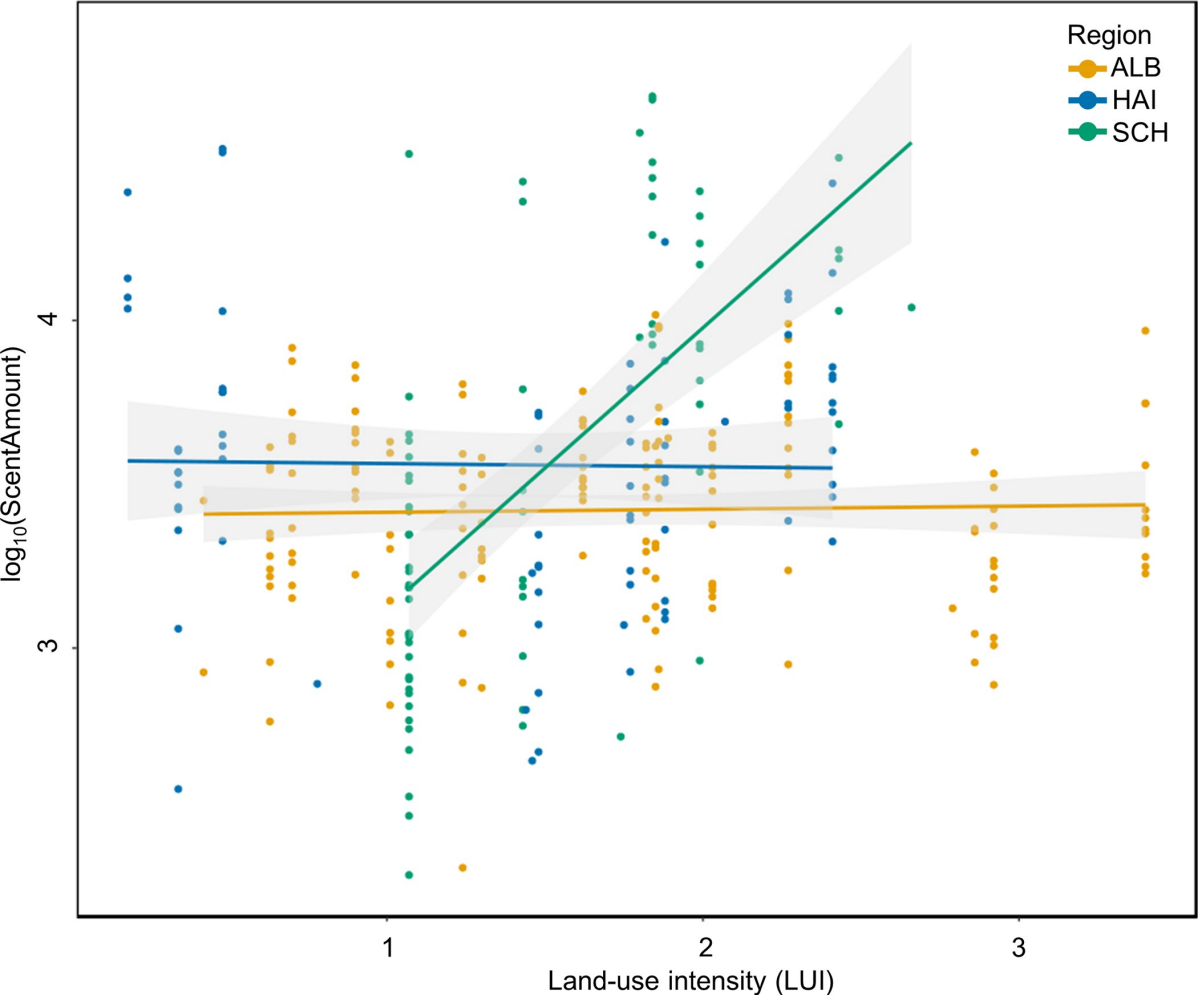
Effects of land-use intensity index LUI and of region on scent amount of *B*. *lapidarius* workers. LUI alone had no effect on scent amount, but an interaction occurred between LUI and region (LMM: χ2 = 11.613, N = 307, p < 0.01). Regression lines for each region, namely Schwäbische Alb (ALB), Hainich-Dün (HAI), and Schorfheide-Chorin (SCH), and 95% confidence intervals are shown.

An analysis of the absolute scent amount of *B*. *lapidarius* workers revealed that the cuticular scent amount was not affected by LUI (LMM: χ^2^ = 1.589, N = 307, p = 0.208). We observed a trend that region affected the total scent amount of bumble bee workers; however, the difference was not significant (LMM: χ^2^ = 4.851, N = 307, p = 0.088). An interaction of region and LUI was shown to affect scent amount significantly (LMM: χ^2^ = 11.613, N = 307, p < 0.01; Fig 3). Since there was no difference but at least a trend for region, we analyzed each region separately for LUI-induced effects on the scent amount of bumble bees. No effect of LUI on scent amount was detected in the Schwäbische Alb (LMM: χ^2^ = 0.022, N = 158, p = 0.882) or in the Hainich-Dün (LMM: χ^2^ = 0, N = 76, p = 0.996), but a strong effect was noted in the Schorfheide-Chorin (LMM: χ^2^ = 5.904, N = 73, p < 0.01; Fig 3).

### Effects on body size

**Fig 4:**
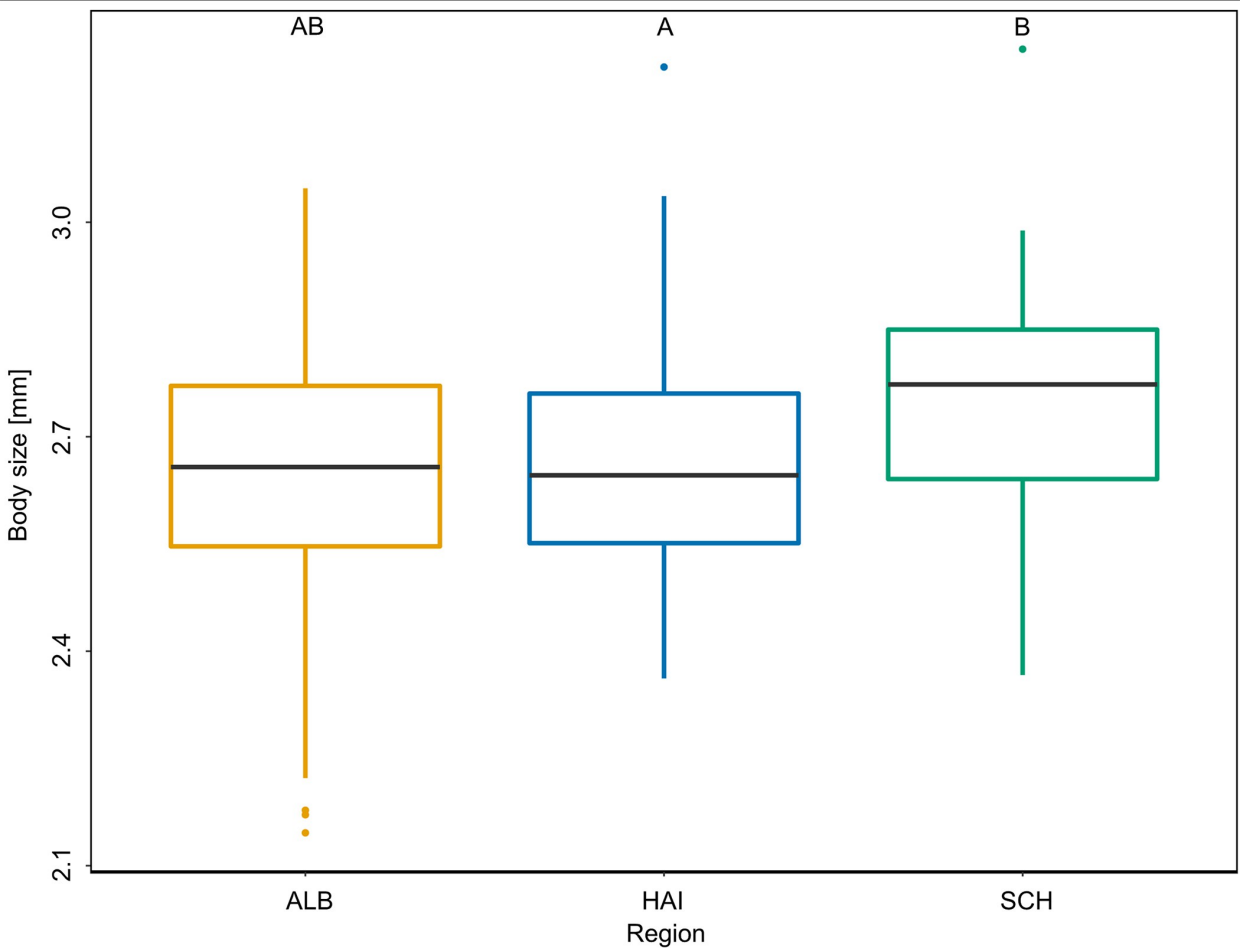
Comparison of body size of *B*. *lapidarius* workers in the various sampling regions. Body size of workers significantly differed in the three regions, namely Schwäbische Alb (ALB), Hainich-Dün (HAI), and Schorfheide-Chorin (SCH) (LMM: χ2 = 6.682, N = 307, p < 0.05). Boxplots show the median range, interquartile range, and the minimum and maximum ranges. Outliers are shown as individual dots. Different capital letters indicate significant differences among groups.

**Fig 5:**
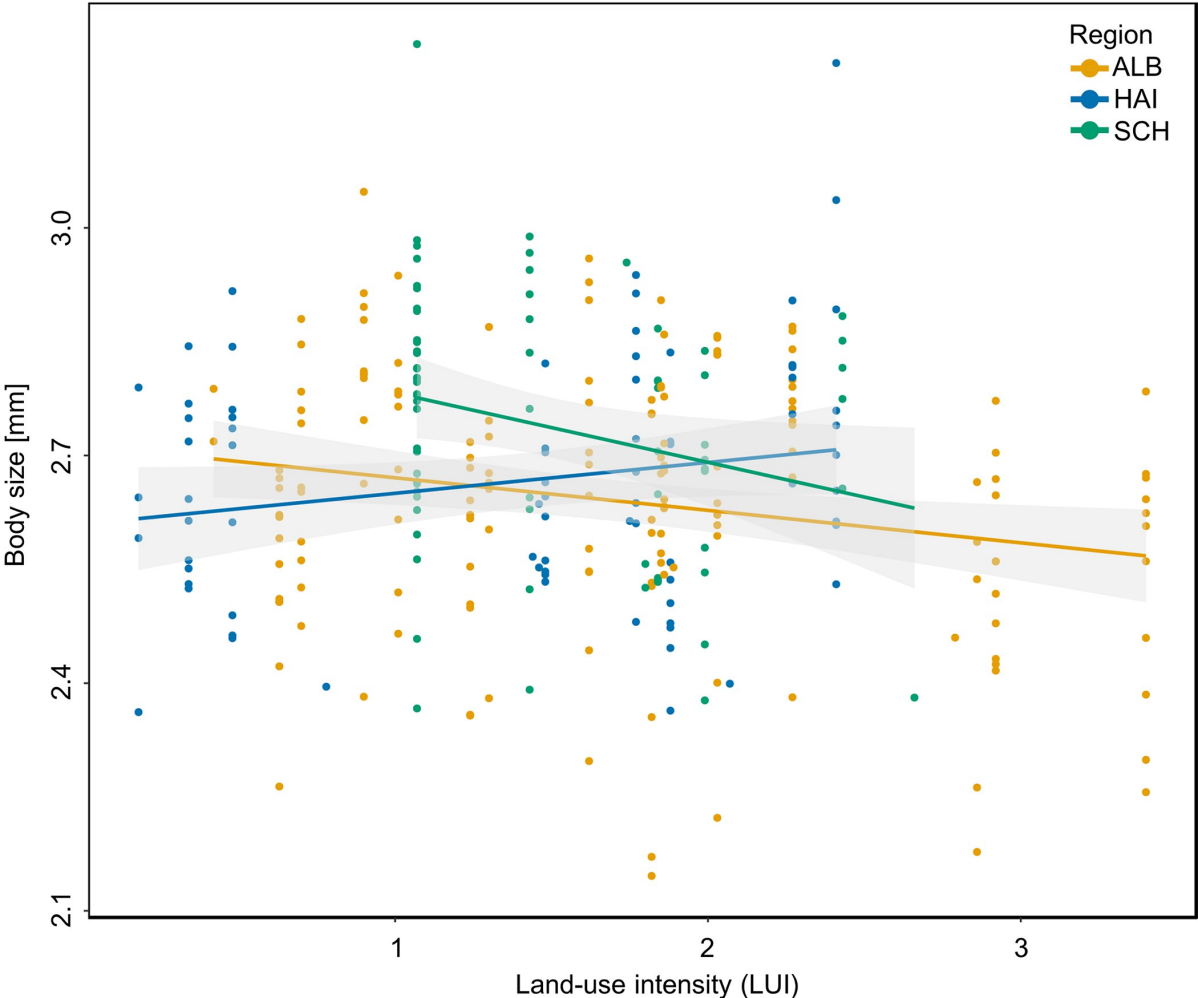
Effects of land-use intensity index LUI and of region on body size of *B*. *lapidarius* workers. The decreasing body size of workers can be explained by an interaction of LUI and region (LMM: χ2 = 6.682, N = 307, p < 0.05). Regression lines for each region, namely Schwäbische Alb (ALB), Hainich-Dün (HAI), and Schorfheide-Chorin (SCH), and 95% confidence intervals are shown.

In our analysis, differences in body size could not be explained by land-use intensity LUI (LMM: χ^2^ = 1.997, N = 307, p = 0.158). The body size of *B*. *lapidarius* workers significantly differed between the three study regions (LMM: χ^2^ = 6.682, N = 307, p < 0.05; Fig 4). A post-hoc test for region revealed significant differences between the regions Schorfheide-Chorin and Hainich-Dün (Post-hoc Tukey-test: p < 0.05). Furthermore, an interaction of LUI and region was seen, with significantly smaller individuals on increasing LUI (LMM: χ^2^ = 7.095, N = 307, p < 0.05; Fig 5). This effect was stronger than the effect of region alone.

## Discussion

In our study, plot, LUI, and ambient air temperature significantly affected the cuticular scent profile of *B*. *lapidarius* workers with plot identity being the most important variable. Further, the total amount of scent as well as body size were affected by an interaction of land-use intensity and region.

### Scent profile of *B*. *lapidarius*

Our analysis of the scent profile in *B*. *lapidarius* workers showed changes attributable to plot, land-use intensity, and temperature. A region-specific effect of LUI was also seen on the total amount of scent on the cuticle surface. The plot-specific scent bouquet of bumble bees might be a result of the presence of different bee colonies on the different EPs in which we collected the bees. In this case, plot could be a proxy for colony, since we considered bees on each EP to derive from plot-specific colonies, and *B*. *lapidarius* is known to show such colony-specific scent profiles [63]. Genetic distinctness might also have played a role in the differences identified. Populations are known to vary in their chemical scent profile and to have population-specific dialects [50, 51]. In isolated populations, genetic drift and/or divergent selection and adaption to local habitat conditions in the three distinct regions might have been the reason for such genetic distinctness and the recorded differences in cuticular hydrocarbons [64, 65]. Furthermore, the CHC profile was found to be prone to changes. Pesticide-induced stress in the German cockroach and bumble bees have been shown to result in changes in the CHC bouquet [66, 67]. Hence, the differences in the chemical surface profile of our bees either are the result of sampling from the different colonies or might be induced by stress related to land-use and land management.

With regard to CHC synthesis, small changes in scent bouquet can result from the incorporation of dietary scent compounds into the lipid profile of an insect, as shown for the grasshopper [68]. Changes in the CHC profile of insects have indeed been shown to result from differences in the quality and quantity of their diet [69]. Insects are generally accepted to synthesize the majority of their hydrocarbons themselves, by elongating precursor compounds such as fatty acids that derived from their diet [40, 70]. Thus, the differences in the CHC profiles of our bees might be attributable to the sampling plots varying in their land-use intensity and thus in their floral diversity and pollen quality [8].

The alkane pentacosane showed the highest loadings in CAP1 and CAP2 and thus contributed most to explaining the variation of scent profiles. In bumble bees, egg-laying is performed mostly by the queen who uses a queen pheromone to suppress ovary development in the workers. Pentacosane, a highly conserved queen pheromone, prevents workers from laying unfertilized eggs [71]. Thus, the maintenance of the dominance of the queen is important in colony success in bees. Furthermore, pentacosane significantly increases in workers at the competition point (the point at which worker reproduction starts and is no longer supressed by the queen) of a colony and thus signals this turning point and the loss of the queen’s dominance [48]. Disruption to the regulation of reproduction might therefore have dramatic effects on colony maintenance and might result in smaller colony sizes.

Hentriacontane also showed a high loading in CAP1. The alkane hentriacontane, together with heneicosane, has been demonstrated to be important in the discrimination of workers from different colonies [72]. We caught workers on several EPs and considered them to be derived from plot-specific colonies, each with its colony-specific scent. The importance of hentriacontane for the discrimination of workers from different colonies might further explain the differences that we have detected in scent bouquet. If the variation in scent profile, and especially in hentriacontane, is too high, then nestmates will no longer be recognized as such and will not be allowed into their own colony. Colony success will therefore suffer over time, and colony maintenance will be disrupted.

The scent profile of CHCs was also found to be affected by desiccation stress and air temperature [40, 42, 44]. Insects are able to adjust and alter their chemical profile on the cuticle surface rapidly in order to cope with new climatic conditions, such as higher temperatures leading to reduced humidity [42, 44]. Changes in the chemical profile represent quantitative changes either in certain CHC classes or in the total amount of cuticular hydrocarbons. In our study, we found a region-specific effect of increasing LUI, which resulted in higher amounts of CHCs in the Schorfheide-Chorin region, whereas no interaction of region and LUI was detected in the remaining two regions. Thus, the significant interaction of region and LUI that we registered across all regions might have been the result of a very strong effect, which was probably driven only by the Schorfheide-Chorin region. Temperature had no effect in the actual model, although temperature in the Schorfheide-Chorin was significantly higher compared with that in Hainich-Dün or in the Schwäbische Alb (S1 Fig). Our results of increasing amounts of CHCs can be interpreted as a response to desiccation stress in the Schorfheide-Chorin region.

### Effects on size of *B*. *lapidarius*

Bumble bee body size differed in our three study regions and decreased with increasing LUI, although the latter was a regional effect. Decreasing body size can have various causes: alloethism and food provisioning. Since we only sampled individuals that were foraging on plants, the differences in size are unlikely to be explained by alloethism. The differences in body size that we have found are more likely to be related to food provisioning during larval development, as shown by former studies in which smaller workers occur as a result of limited food availability [29–31]. Interestingly, several investigations performed in the Biodiversity Exploratories have found a decrease in floral abundance and diversity, which result in a concomitant decrease in pollen quantity and diversity, in grassland sites with increasing land-use intensity [6, 8]. Both pollen quantity and pollen quality have been shown to affect body size in sweat bees [73]. The smaller individuals that we have found in the high intensity plots in our study are thus highly likely to be the result of the lower pollen quantity in high intensity plots or in the surrounding landscape. Various other factors, in addition to the availability of food, have been described to affect body size. Bumble bees exposed to natural stressors such as toxin stress, parasite stress, and temperature stress differ in their wing size [74], which is the same proxy measurement that we have used for body size in our study.

Amongst its various possible effects, smaller body size can influence foraging and the reproductive success of colonies. Several studies have shown that bigger individuals have an advantage in terms of thermoregulation, and hence they can forage even at cooler temperatures [35, 38]. Larger individuals also have an advantage in terms of foraging distance, since they have stronger flight muscles compared with smaller bees. They can thus forage over greater distances and find new and isolated highly rewarding habitats more easily in fragmented low-quality areas [33, 34, 36, 75]. For smaller individuals with a smaller foraging range, these areas remain inaccessible with subsequent effects on the colony attributable to food restriction. Furthermore, larger individuals have been shown to carry bigger forage loads and are more likely to forage for nectar [31, 33]. With smaller individuals carrying smaller amounts of food and the amount of food provisioning during larval development being directly linked to body size, the colony success of bumble bees seems to be linked to their body size [76]. Taken together, our results support the hypothesis that smaller bees are the result of poor quality of food and are, in turn, less effective in foraging, thereby leading to decreased colony success.

## Conclusion

Our results clearly show that the chemical profile of the cuticle surface in *B*. *lapidarius* is affected by temperature and land-use intensity. Since surface compounds have a key function in intracolonial chemical communication in social insects, changes in their chemical profile can have strong effects on the stability of a colony, colony maintenance, and reproductive success. Further, we found that the body size of our bumble bees is influenced by a region-specific effect of land-use intensity. Since body size directly affects foraging success and foraging behavior, it probably also affects colony maintenance and reproduction as a consequence. Taken together, our results support the assumption that the intensification of land-use and an increase in temperature affect important pollinators and can contribute to ongoing insect loss. Changes in the chemical profile of *B*. *lapidarius* can cause disruption in their colony structure and lead, for example, to a loss of queen dominance.

Since we have not finally clarified which of the examined stressors are responsible for the changes in scent bouquet and size that we have detected, additional studies should be performed with the aim of disentangling the effect of pesticides, low diet, and other stressors. Since pollinators and, in our case, bumble bees are mobile insects, the effects of the plots, each of which is surrounded with different landscape elements, should also be considered in future studies. To the best of our knowledge, we have shown, for the first time, that land-use intensity induces changes in the chemical profile and causes a decrease in body size in a common bumble bee species, *Bombus lapidarius*, in Europe.

## Supporting information

S1 Table List of all EPs in the three regions (ALB = Schwäbische Alb, HAI = Hainich-Dün, SCH = Schorfheide-Chorin) with grazing, mowing and fertilization as well as their land-use intensity (LUI) and the resulting land-use type. (DOCX)

S2 Table List of all 49 chemical substances analyzed for each individual bumble bee worker. (DOCX)

S3 Table Loadings of CAP1 and CAP2 obtained from the db-RDA. Compounds that had the highest loadings for CAP1 and CAP2 and that contributed most to the separation are marked in bold.
(DOCX)

S1 Fig Gas chromatogram of cuticular surface compounds analyzed for each *B*. *lapidarius* worker. The gas chromatogram shows the peaks of all 49 analyzed compounds listed in S2 Table.
(TIF)

S2 Fig Comparison of ambient air temperature in the three different study regions. Ambient air temperature significantly differed between regions (LMM: χ2 *=* 37.70, p < 0.001). Region Schorfheide-Chorin (SCH) differed significantly from regions Schwäbische Alb (ALB, Post-hoc Tukey-test: p < 0.001) and Hainich-Dün (HAI, Post-hoc Tukey-test: p < 0.05). Region Schwäbische Alb (ALB) differed significantly from region Hainich-Dün (HAI, Post-hoc Tukey-test: p < 0.001). Boxplots show the median range, interquartile range, and the minimum and maximum ranges. Different capital letters indicate significant differences among groups.
(TIF)
