# Supplementary material for: Land-use stress alters cuticular chemical surface profile and morphology in the bumble bee Bombus lapidarius
Source: PLoS One. 2022 May 13;17(5):e0268474. doi: 10.1371/journal.pone.0268474 (PMC9106155; doi:10.1371/journal.pone.0268474)
Supplement: S1 Table — (DOCX) [file pone.0268474.s001.docx]

**Tab S1:** **List of all EPs in the three regions (ALB = Schwäbische Alb, HAI = Hainich-Dün, SCH = Schorfheide-Chorin) with grazing, mowing and fertilization as well as their land-use intensity (LUI) and the resulting land-use type.**

| Region | Plot | Grazing | Mowing | Fertilization | LUI | Land-use type |
| --- | --- | --- | --- | --- | --- | --- |
| ALB | AEG1 | 0 | 2 | 74.7 | 2.03 | Meadow, fertilized |
| ALB | AEG12 | 0 | 2 | 45 | 1.82 | Meadow, fertilized |
| ALB | AEG14 | 0 | 3 | 169.5 | 2.79 | Meadow, fertilized |
| ALB | AEG15 | 0 | 3 | 304 | 3.4 | Meadow, fertilized |
| ALB | AEG21 | 624.1 | 1 | 72.1 | 2.92 | Mown pasture, fertilized |
| ALB | AEG22 | 0 | 1 | 0 | 1.01 | Meadow, unfertilized |
| ALB | AEG24 | 6.4 | 3 | 182.1 | 2.86 | Mown pasture, fertilized |
| ALB | AEG25 | 45.6 | 0 | 0 | 0.63 | Pasture, unfertilized |
| ALB | AEG29 | 182.6 | 1 | 0 | 1.62 | Mown pasture, unfertilized |
| ALB | AEG30 | 76.1 | 1 | 0 | 1.3 | Mown pasture, unfertilized |
| ALB | AEG32 | 19.6 | 0 | 0 | 0.42 | Pasture, unfertilized |
| ALB | AEG34 | 58 | 1 | 0 | 1.24 | Mown pasture, unfertilized |
| ALB | AEG41 | 13.4 | 2 | 107.8 | 2.27 | Meadow, fertilized |
| ALB | AEG45 | 0 | 3 | 17.6 | 1.89 | Meadow, unfertilized |
| ALB | AEG46 | 388.1 | 0 | 0 | 1.85 | Pasture, unfertilized |
| ALB | AEG48 | 55.1 | 0 | 0 | 0.7 | Pasture, unfertilized |
| ALB | AEG50 | 0 | 3 | 14.7 | 1.86 | Meadow, unfertilized |
| ALB | AEG7 | 91 | 0 | 0 | 0.9 | Pasture, unfertilized |
| HAI | HEG17 | 26.1 | 0 | 0 | 0.48 | Pasture, unfertilized |
| HAI | HEG26 | 95.3 | 1 | 117.6 | 2.27 | Mown pasture, fertilized |
| HAI | HEG29 | 22.7 | 1 | 66 | 1.75 | Mown pasture, fertilized |
| HAI | HEG32 | 125.5 | 1 | 50 | 1.88 | Mown pasture, fertilized |
| HAI | HEG34 | 4.6 | 1 | 170.6 | 2.41 | Mown pasture, fertilized |
| HAI | HEG38 | 71.5 | 1 | 15.1 | 1.44 | Mown pasture, fertilized |
| HAI | HEG42 | 3.6 | 0 | 0 | 0.18 | Pasture, unfertilized |
| HAI | HEG43 | 13.3 | 0 | 0 | 0.34 | Pasture, unfertilized |
| HAI | HEG48 | 0 | 1 | 75.6 | 1.77 | Meadow, fertilized |
| HAI | HEG49 | 0 | 1 | 42 | 1.48 | Meadow, unfertilized |
| HAI | HEG50 | 0 | 1 | 39.3 | 1.46 | Meadow, fertilized |
| HAI | HEG6 | 4.6 | 1 | 170.6 | 2.41 | Mown pasture, fertilized |
| HAI | HEG9 | 69.1 | 0 | 0 | 0.78 | Pasture, unfertilized |
| SCH | SEG17 | 335.3 | 1 | 0 | 1.99 | Mown pasture, unfertilized |
| SCH | SEG18 | 0 | 2 | 0 | 1.43 | Meadow, unfertilized |
| SCH | SEG20 | 266.7 | 1 | 0 | 1.84 | Mown pasture, unfertilized |
| SCH | SEG21 | 253.7 | 1 | 0 | 1.8 | Mown pasture, unfertilized |
| SCH | SEG25 | 0 | 2 | 0 | 1.43 | Meadow, unfertilized |
| SCH | SEG30 | 15 | 1 | 0 | 1.07 | Mown pasture, unfertilized |
| SCH | SEG31 | 15 | 1 | 0 | 1.07 | Mown pasture, unfertilized |
| SCH | SEG32 | 15 | 1 | 0 | 1.07 | Mown pasture, unfertilized |
| SCH | SEG35 | 181.9 | 0 | 50.8 | 1.74 | Pasture, fertilized |
| SCH | SEG38 | 803.8 | 0 | 0 | 2.66 | Pasture, unfertilized |
| SCH | SEG48 | 440.6 | 2 | 0 | 2.43 | Mown pasture, unfertilized |
