# Supplementary material for: Land-use stress alters cuticular chemical surface profile and morphology in the bumble bee Bombus lapidarius
Source: PLoS One. 2022 May 13;17(5):e0268474. doi: 10.1371/journal.pone.0268474 (PMC9106155; doi:10.1371/journal.pone.0268474)
Supplement: S2 Table — (DOCX) [file pone.0268474.s002.docx]

**Tab S2:** **List of all 49 chemical substances analyzed for each individual bumble bee worker.**

| **ID** | **Scent compound** |  | **ID** | **Scent compound** |
| --- | --- | --- | --- | --- |
| 1 | Nonadecane |  | 26 | Nonacosane |
| 2 | Eicosane |  | 27 | Tricontane |
| 3 | (Z)-9-Heineicosene |  | 28 | Hentriacontene 1* |
| 4 | (Z)-7-Heneicosene |  | 29 | Hentriacontene 2* |
| 5 | Heineicosane |  | 30 | Hentriacontane |
| 6 | Docosane |  | 31 | Tetradecyl hexadecanoate |
| 7 | (Z)-11-Tricosene |  | 32 | Dotriacontane |
| 8 | (Z)-9-Tricosene |  | 33 | Tritriacontene 1* |
| 9 | (Z)-7-Tricosene |  | 34 | Tritriacontene 2* |
| 10 | (Z)-5-Tricosene |  | 35 | Tritriacontane |
| 11 | Tricosane |  | 36 | Hexadecyl hexadecanoate |
| 12 | Tetracosane |  | 37 | Tetratriacontane |
| 13 | (Z)-11-Pentacosene |  | 38 | Pentatriacontane |
| 14 | (Z)-9-Pentacosene |  | 39 | Octadecyl hexadecanoate |
| 15 | (Z)-7-Pentacosene |  | 40 | Eicosyl hexadecanoate |
| 16 | (Z)-5-Pentacosene |  | 41 | Eicosyloleate |
| 17 | Pentacosane |  | 42 | Eicosyl octadecanoate |
| 18 | Hexacosane |  | 43 | Docosyl hexadecanoate |
| 19 | (Z)-11-Heptacosene |  | 44 | Docosyloleate |
| 20 | (Z)-9-Heptacosene |  | 45 | Docosyl octadecanoate |
| 21 | (Z)-7-Heptacosene |  | 46 | Tetracosyloleate |
| 22 | Heptacosane |  | 47 | Tetracosyl octadecanoate |
| 23 | Octacosane |  | 48 | Hexacosyloleate |
| 24 | (Z)-9-Nonacosene |  | 49 | Hexacosyl octadecanoate |
| 25 | (Z)-7-Nonacosene |  |  |  |
