# Supplementary material for: Land-use stress alters cuticular chemical surface profile and morphology in the bumble bee Bombus lapidarius
Source: PLoS One. 2022 May 13;17(5):e0268474. doi: 10.1371/journal.pone.0268474 (PMC9106155; doi:10.1371/journal.pone.0268474)
Supplement: S3 Table — Compounds that had the highest loadings for CAP1 and CAP2 and that contributed most to the separation are marked in bold. (DOCX) [file pone.0268474.s003.docx]

**Tab S3:** **Loadings of CAP1 and CAP2 obtained from the db-RDA.** Compounds that had the highest loadings for CAP1 and CAP2 and that contributed most to the separation are marked in bold.

| **Scent compound** | **CAP1** | **CAP2** |  | **Scent compound** | **CAP1** | **CAP2** |
| --- | --- | --- | --- | --- | --- | --- |
| Nonadecane | 0.007 | -0.005 |  | Nonacosane | 0.780 | -0.203 |
| Eicosane | 0.001 | 0.004 |  | Tricontane | 0.071 | -0.054 |
| (Z)-9-Heineicosene | -0.002 | -0.001 |  | Hentriacontene 1* | -0.122 | -0.069 |
| (Z)-7-Heneicosene | -0.019 | -0.008 |  | Hentriacontene 2* | -0.300 | -0.167 |
| Heineicosane | 0.086 | -0.179 |  | **Hentriacontane** | **1.105** | -0.831 |
| Docosane | -0.007 | -0.007 |  | Tetradecyl hexadecanoate | 0.004 | -0.008 |
| (Z)-11-Tricosene | -0.012 | -0.002 |  | Dotriacontane | 0.120 | -0.136 |
| (Z)-9-Tricosene | -0.065 | 0.030 |  | Tritriacontene 1* | -0.034 | -0.027 |
| (Z)-7-Tricosene | -0.808 | -0.134 |  | Tritriacontene 2* | -0.043 | -0.064 |
| (Z)-5-Tricosene | -0.042 | -0.001 |  | Tritriacontane | 0.226 | -0.311 |
| Tricosane | -0.757 | 0.449 |  | Hexadecyl hexadecanoate | 0.017 | -0.015 |
| Tetracosane | 0.007 | 0.057 |  | Tetratriacontane | 0.058 | -0.067 |
| (Z)-11-Pentacosene | -0.023 | -0.001 |  | Pentatriacontane | 0.027 | -0.030 |
| (Z)-9-Pentacosene | -0.354 | 0.045 |  | Octadecyl hexadecanoate | 0.017 | -0.010 |
| **(Z)-7-Pentacosene** | **-2.454** | 0.180 |  | Eicosyl hexadecanoate | 0.020 | -0.002 |
| (Z)-5-Pentacosene | 0.036 | -0.164 |  | Eicosyloleate | 0.032 | 0.017 |
| **Pentacosane** | **1.013** | **1.840** |  | Eicosyl octadecanoate | 0.010 | 0.004 |
| Hexacosane | 0.014 | -0.002 |  | Docosyl hexadecanoate | 0.013 | 0.002 |
| (Z)-11-Heptacosene | -0.003 | 0.001 |  | Docosyloleate | 0.019 | -0.014 |
| (Z)-9-Heptacosene | -0.018 | -0.015 |  | Docosyl octadecanoate | 0.061 | 0.062 |
| (Z)-7-Heptacosene | -0.315 | -0.131 |  | Tetracosyloleate | 0.750 | -0.545 |
| Heptacosane | 0.185 | -0.083 |  | Tetracosyl octadecanoate | 0.272 | 0.296 |
| Octacosane | 0.022 | -0.015 |  | Hexacosyloleate | 0.475 | 0.416 |
| (Z)-9-Nonacosene | -0.009 | -0.026 |  | Hexacosyl octadecanoate | 0.176 | 0.039 |
| (Z)-7-Nonacosene | -0.239 | -0.113 |  |  |  |  |
