# Supplementary figures and images for: Land-use stress alters cuticular chemical surface profile and morphology in the bumble bee Bombus lapidarius
Source: PLoS One. 2022 May 13;17(5):e0268474. doi: 10.1371/journal.pone.0268474 (PMC9106155; doi:10.1371/journal.pone.0268474)

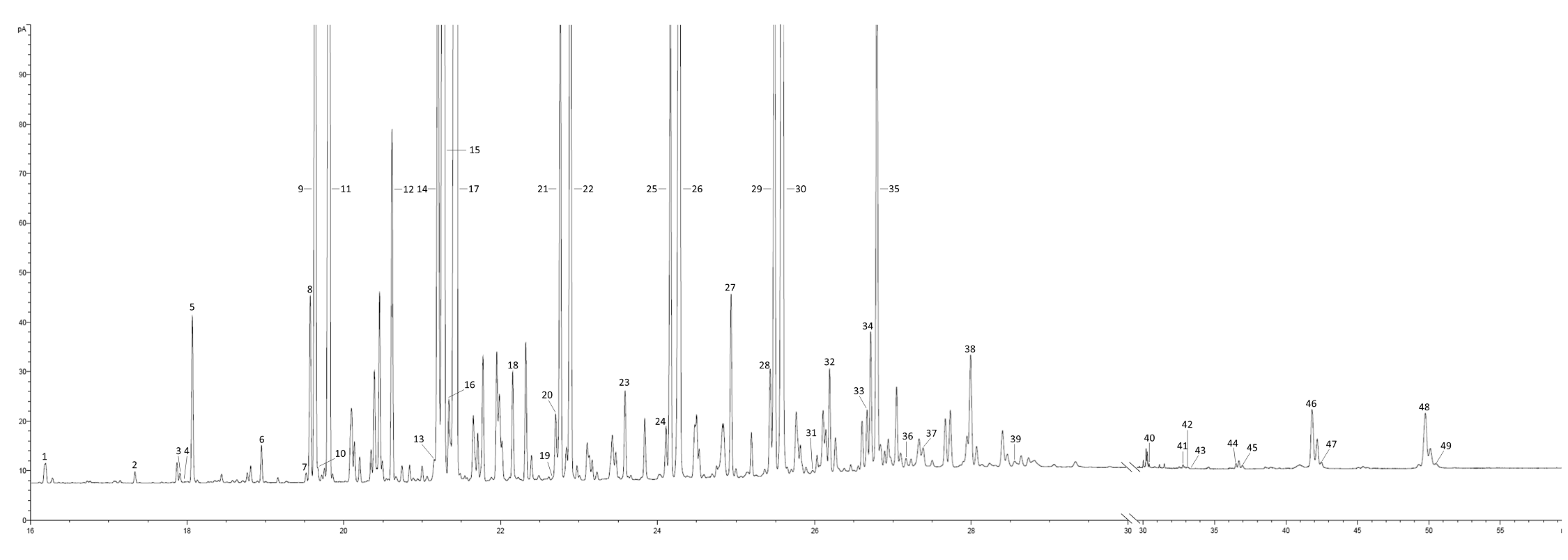

Supplement: S1 Fig — The gas chromatogram shows the peaks of all 49 analyzed compounds listed in S2 Table. (TIF) [file pone.0268474.s004.tif]

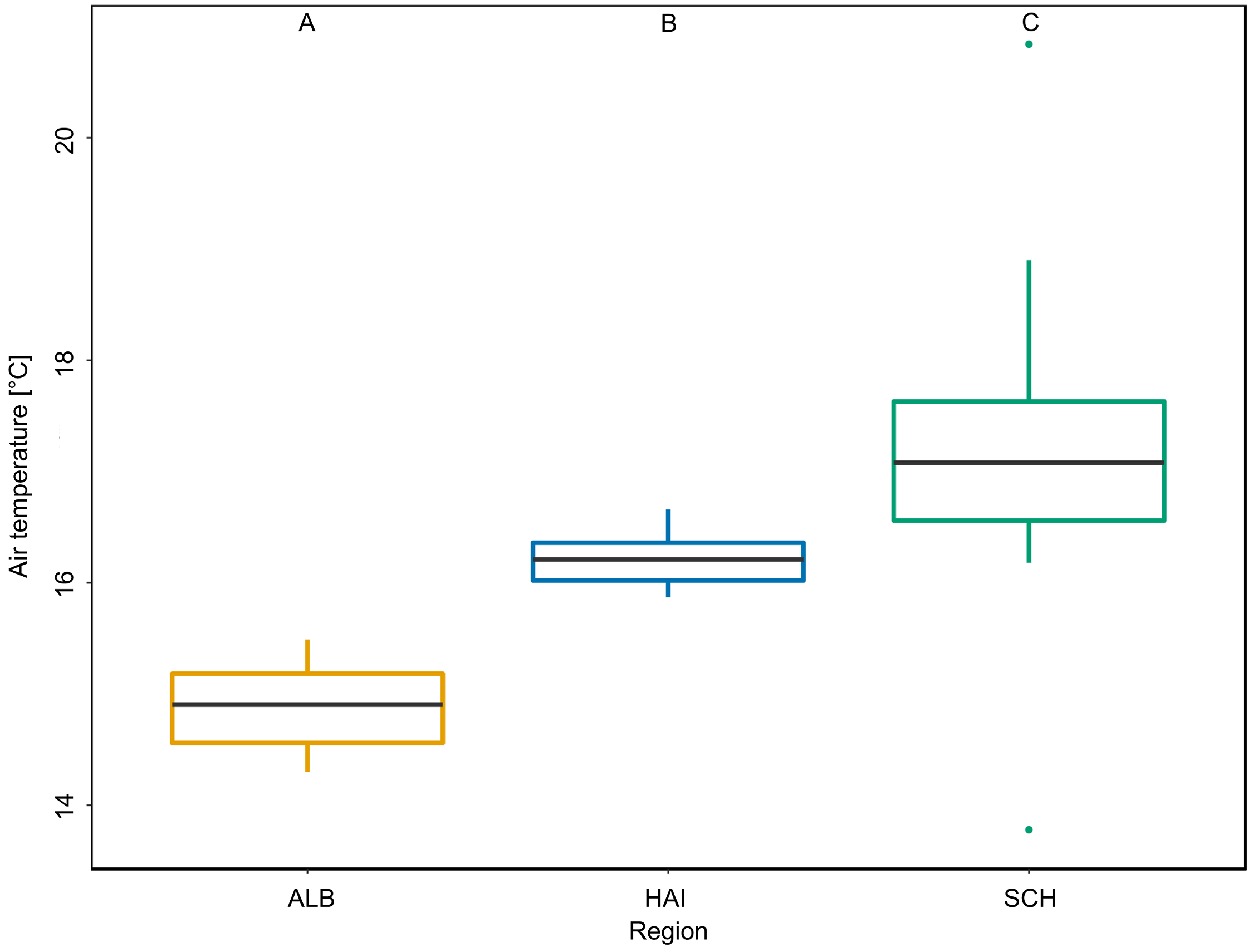

Supplement: S2 Fig — Ambient air temperature significantly differed between regions (LMM: χ2 = 37.70, p < 0.001). Region Schorfheide-Chorin (SCH) differed significantly from regions Schwäbische Alb (ALB, Post-hoc Tukey-test: p < 0.001) and Hainich-Dün (HAI, Post-hoc Tukey-test: p < 0.05). Region Schwäbische Alb (ALB) differed significantly from region Hainich-Dün (HAI, Post-hoc Tukey-test: p < 0.001). Boxplots show the median range, interquartile range, and the minimum and maximum ranges. Different capital letters indicate significant differences among groups. (TIF) [file pone.0268474.s005.tif]
